# Diagnostic efficacy of contrast-enhanced fluid-attenuated inversion recovery (FLAIR) imaging in idiopathic cerebrospinal fluid rhinorrhea

**DOI:** 10.1016/j.radcr.2024.07.018

**Published:** 2024-07-26

**Authors:** Hiroaki Watanabe, Hidetoshi Arai, Kazuho Ogihara, Hiroyuki Morisaka, Akitoshi Saito, Motohiro Moriyama, Shin Nakano, Kenta Aonuma, Kaori Aoyagi, Keiko Matsumoto, Keiji Toyama, Hiroshi Onishi

**Affiliations:** a Department of Radiology, Yamanashi Central Hospital, Kofu, Yamanashi, Japan; b Department of Radiology, University of Yamanashi, Chuo, Yamanashi, Japan; c Department of Otorhinolaryngology and Head & Neck Surgery, Yamanashi Central Hospital, Kofu, Yamanashi, Japan; d Department of Neurosurgery, Yamanashi Central Hospital, Kofu, Yamanashi, Japan

**Keywords:** CSF rhinorrhea, CE-FLAIR, MR cisternography, Case report

## Abstract

We report a case of a 50-year-old woman in which contrast-enhanced fluid-attenuated inversion recovery (FLAIR) was used for the diagnosis of idiopathic cerebrospinal fluid rhinorrhea. The pre- and postcontrast FLAIR subtraction images showed a contrasted protrusion of the right olfactory cleft canal, highlighting the potential practicality and effectiveness of using pre- and postcontrast FLAIR subtraction images in diagnosing idiopathic cerebrospinal fluid rhinorrhea, in conjunction with conventional high-resolution computed tomography and magnetic resonance cisternography. The successful diagnosis of cerebrospinal fluid rhinorrhea allowed for treatment through endoscopic nasal surgery to close the fistula with a positive clinical outcome.

## Introduction

High-resolution computed tomography (CT) and magnetic resonance (MR) cisternography are conventionally used for imaging diagnosis of idiopathic cerebrospinal fluid (CSF) rhinorrhea [1,2]. Contrast-enhanced MR cisternography has also been reported to be beneficial for CSF rhinorrhea diagnosis [[3], [4], [5]]. However, CT may be unable to identify small bone defects [6]. There is also the limitation that even if a bone defect can be identified, it is not necessarily complicated by a dural release [2,7]. MR cisternography has been reported to have high sensitivity and specificity (80%-90%) for the diagnosis of CSF rhinorrhea but is inferior to contrast-enhanced MR cisternography [4,5]. Contrast-enhanced MR cisternography has high diagnostic performance but requires intrathecal contrast administration and is an invasive test. On the other hand, postcontrast FLAIR has been reported to be useful in diagnosing lesions bordering the CSF [8] but has yet to be evaluated as a potential tool in diagnosing CSF rhinorrhea. Here, we report a case in which a pre- and postcontrast FLAIR thin slice subtraction image, combined with high-resolution CT and MR cisternography, was used to diagnose CSF rhinorrhea.

### Case report

**Fig. 1:**
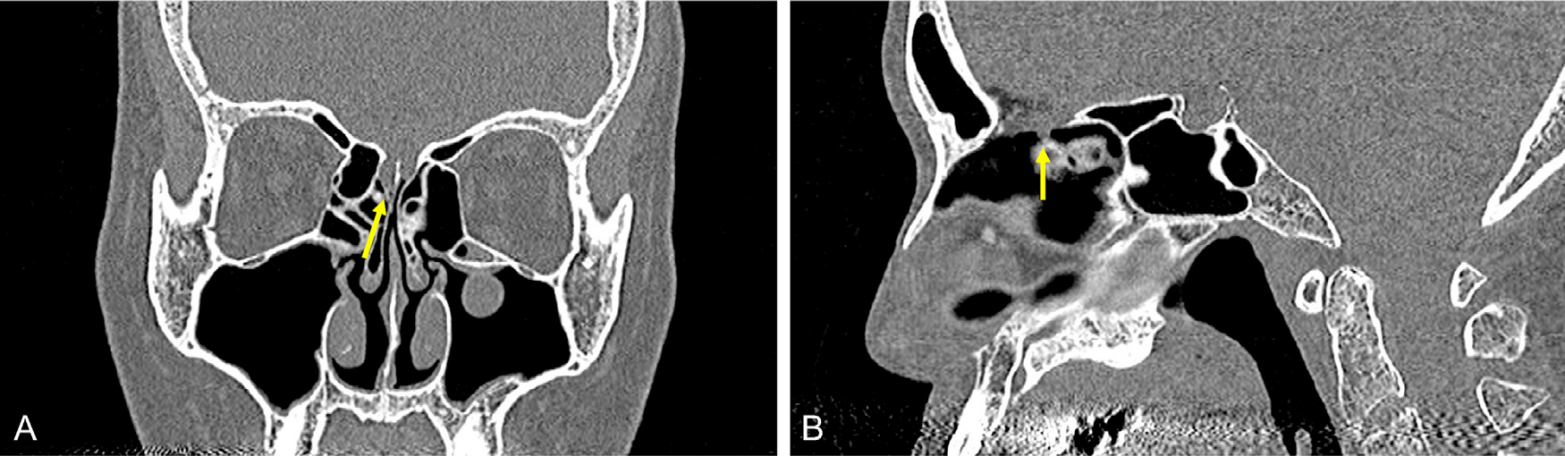
Reconstruction of the initial CT with a 0.5-mm slice. The coronal image (A) and sagittal image (B) of the CT reveal a protrusion of soft density from the right ethmoid cribriform plate toward the nasal cavity (arrow).

**Fig. 2:**
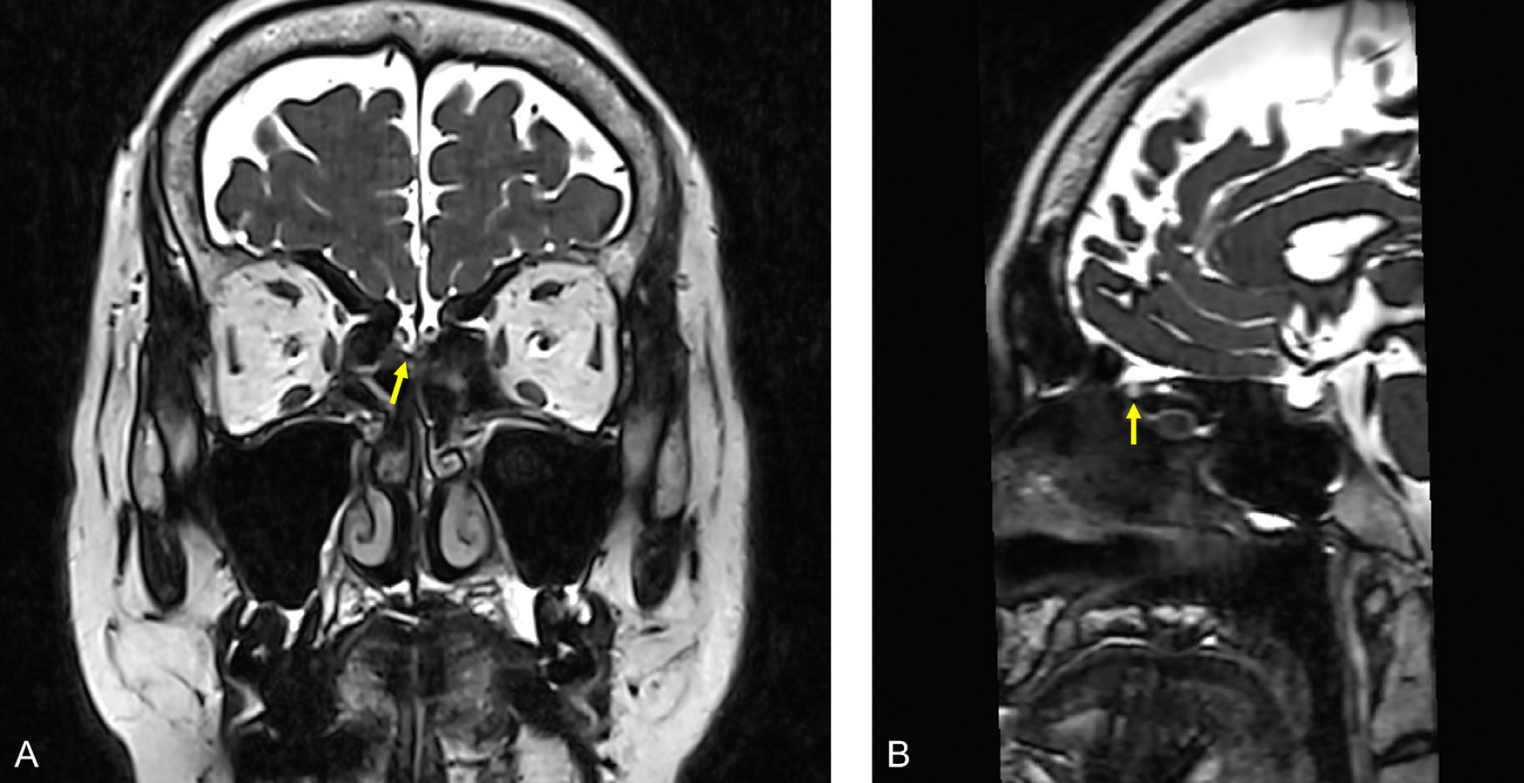
MR cisternography demonstrates pooling of CSF in the right nasal cavity due to the defect in the right cribriform plate of the ethmoid bone. However, the findings are minor (A, B, arrow).

**Fig. 3:**
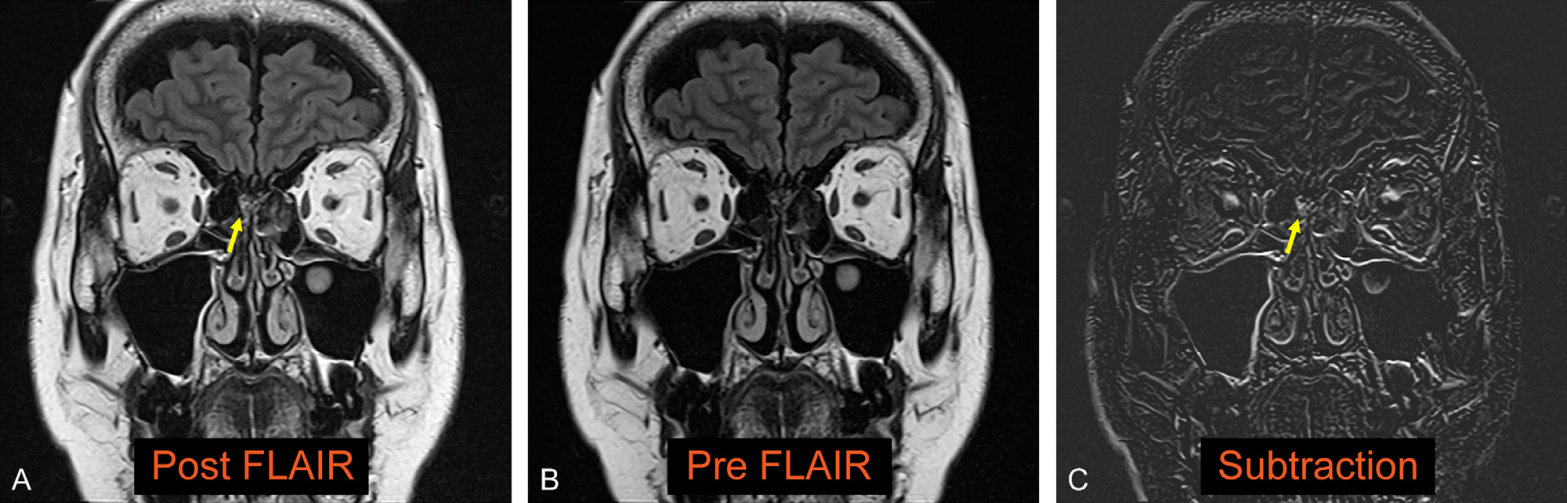
Pre- and postcontrast FLAIR subtraction images show a contrast effect consistent with a protrusion of the right cribriform plate of the ethmoid bone (A-C, arrow).

**Fig. 4:**
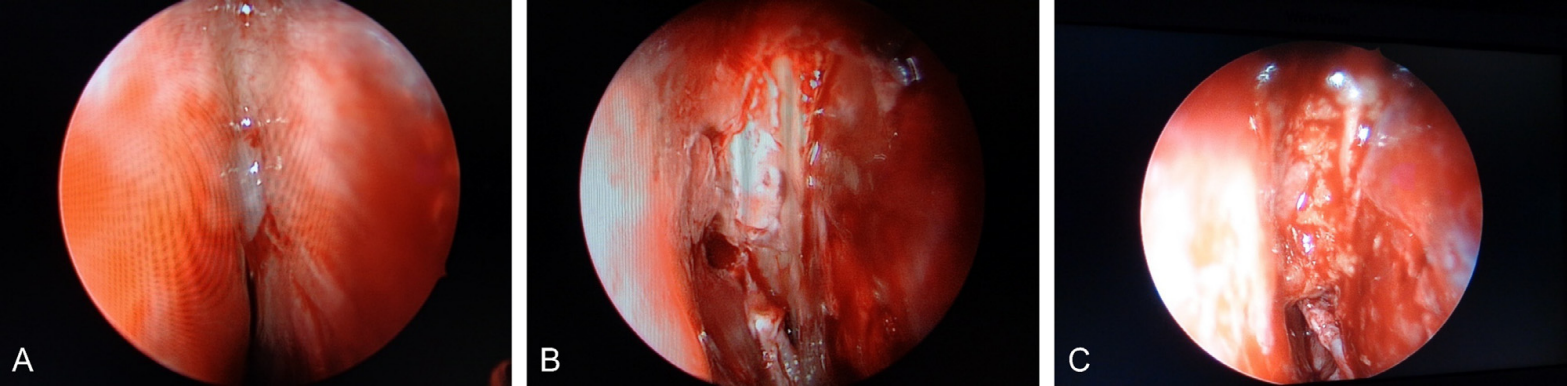
Endoscopic nasal surgery was performed, and a fistula was identified posterior to the olfactory fissure (A). In the same area, a 1.2 cm long bony defect and dural exposure were noted. Spinal fluid leakage was observed through the fistula (B). The fistula was filled with abdominal fat tissue, covered, and fixed (C).

We report a case of persistent watery rhinorrhea in a woman in her 50s. The patient experienced a fever following coronavirus vaccination. Watery rhinorrhea coincided with fever onset. Her previous physician suspected allergic rhinitis and commenced treatment. However, no improvement was observed, and the patient was referred to our otorhinolaryngology department. The nasopharyngeal fiberscope revealed bilateral nasal mucosal swelling and soluble nasal discharge from the right nasal cavity. The 3-mm-slice CT scan showed no sinusitis or CSF rhinorrhea. Based on the above, severe allergic rhinitis was suspected, and drug treatment and posterior nasal nerve transection were performed. However, the persistent right-sided soluble rhinorrhea during forward bending led to reconsideration of CSF rhinorrhea. An intranasal glucose concentration test was performed, which showed a high value of 58 mg/dL (normal range: < 30 mg/dL). CSF rhinorrhea was suspected, and the MR cisternography scan was performed. At that time, the initial CT was also reviewed. A 0.5-mm slice reconstruction of the initial CT before the MRI scan revealed a soft density protrusion of the right cribriform plate of the ethmoid bone (Fig. 1). MR cisternography also demonstrated CSF pooling in the right nasal cavity through the defect in the same area. However, the findings were minor, making it challenging to determine the pathology (Fig. 2). To further clarify whether these findings were responsible for the patient's ongoing watery rhinorrhea, FLAIR 2-mm slice imaging was conducted both pre- and postcontrast to create a subtraction. The FLAIR imaging displayed a clear contrast effect on the periphery of the CSF pooling in the right nasal cavity (Fig. 3). This finding suggested dural disruption and vascular injury at the same site, leading to the diagnosis of CSF rhinorrhea. Endoscopic nasal surgery was performed, and the fistula was closed (Fig. 4). The postoperative course was successful, and no recurrence was observed 6 months after surgery.

## Discussion

FLAIR is a special pulse sequence that uses inversion recovery with a long repetition time (TR) and echo time (TE), as well as an inversion time (TI) that effectively eliminates signals from the CSF [[9], [10], [11]]. Although FLAIR images are T2-weighted images (T2WI), the contrast effect in contrast-enhanced FLAIR is due to the mild T1 shortening effect caused by the long TI. Additionally, FLAIR not only suppresses CSF but also the intravascular signal in normal blood flow, resulting in a more noticeable contrast effect in the lesion [12]. This is a feature not observed in postcontrast T1-weighted images (T1WI). Therefore, it has been reported that contrast-enhanced FLAIR is useful in diagnosing lesions bordering the CSF. In this report, FLAIR is more sensitive to T1 shortening than T1WI at lower gadolinium (Gd) concentrations [8]. However, the hyperintensity lesions observed with contrast-enhanced FLAIR alone may be caused by either T2 lengthening or T1 shortening. Therefore, both pre- and postcontrast imaging are necessary, and pre- and postcontrast subtraction may be useful [13,14]. Based on these reports of contrast-enhanced FLAIR, we applied pre- and postcontrast FLAIR subtraction to detect CSF rhinorrhea. The pronounced differences in contrast observed in contrast-enhanced FLAIR in our case may be due to trace amounts of Gd contrast agent leaking from the dural disruption and vascular injury in the cribriform plate of the ethmoid bone. Currently, there are no reports of CSF rhinorrhea diagnosed using contrast-enhanced FLAIR imaging. High-resolution CT and MR cisternography can only diagnose the morphology and localization of the lesion. Therefore, when the lesion is small, even if there is evidence of CSF pooling into the nasal cavity, the cribriform plate may simply droop toward the nasal cavity without the dural injury complications, making it difficult to distinguish between the two. In such cases, the addition of pre- and postcontrast-enhanced FLAIR imaging may illuminate local damage and contribute to the diagnosis. Here, we demonstrate the use of pre- and postcontrast FLAIR subtraction to diagnose a case of CSF rhinorrhea that was previously ambiguous through high-resolution CT and MR cisternography. This novel approach enabled appropriate treatment and a favorable clinical outcome for the patient.

## Conclusion

In addition to high-resolution CT and MR cisternography, pre- and postcontrast FLAIR subtraction may prove useful in imaging CSF rhinorrhea.

## Patient consent

Written informed consent was obtained for patient information to be published in this article.

## References

[1] Meco C, Oberascher G (2004). Comprehensive algorithm for skull base dural lesion and cerebrospinal fluid fistula diagnosis. Laryngoscope.

[2] Oakley GM, Alt JA, Schlosser RJ, Harvey RJ, Orlandi RR (2016). Diagnosis of cerebrospinal fluid rhinorrhea: an evidence-based review with recommendations. Int Forum Allergy Rhinol.

[3] Goel G, Ravishankar S, Jayakumar PN, Vasudev MK, Shivshankar JJ, Rose D (2007). Intrathecal gadolinium-enhanced magnetic resonance cisternography in cerebrospinal fluid rhinorrhea: road ahead?. J Neurotrauma.

[4] Algin O, Hakyemez B, Gokalp G, Ozcan T, Korfali E, Parlak M (2010). The contribution of 3D-CISS and contrast-enhanced MR cisternography in detecting cerebrospinal fluid leak in patients with rhinorrhoea. Br J Radiol.

[5] Ecin G, Oner AY, Tokgoz N, Ucar M, Aykol S, Tali T (2013). T2-weighted vs. intrathecal contrast-enhanced MR cisternography in the evaluation of CSF rhinorrhea. Acta Radiol.

[6] Manes RP, Ryan MW, Marple BF (2012). A novel finding on computed tomography in the diagnosis and localization of cerebrospinal fluid leaks without a clear bony defect. Int Forum Allergy Rhinol.

[7] Reddy M, Baugnon K (2017). Imaging of cerebrospinal fluid rhinorrhea and otorrhea. Radiol Clin North Am.

[8] Essig M, Knopp MV, Schoenberg SO, Hawighorst H, Wenz F, Debus J (1999). Cerebral gliomas and metastases: assessment with contrast-enhanced fast fluid-attenuated inversion-recovery MR imaging. Radiology.

[9] De Coene B, Hajnal JV, Gatehouse P, Longmore DB, White SJ, Oatridge A (1992). MR of the brain using fluid-attenuated inversion recovery (FLAIR) pulse sequences. AJNR Am J Neuroradiol.

[10] Rydberg JN, Hammond CA, Grimm RC, Erickson BJ, Jack CR, Huston J (1994). Initial clinical experience in MR imaging of the brain with a fast fluid-attenuated inversion-recovery pulse sequence. Radiology.

[11] Hajnal JV, Bryant DJ, Kasuboski L, Pattany PM, De Coene B, Lewis PD (1992). Use of fluid attenuated inversion recovery (FLAIR) pulse sequences in MRI of the brain. J Comput Assist Tomogr.

[12] Fukuoka H, Hirai T, Okuda T, Shigematsu Y, Sasao A, Kimura E (2010). Comparison of the added value of contrast-enhanced 3D fluid-attenuated inversion recovery and magnetization-prepared rapid acquisition of gradient echo sequences in relation to conventional postcontrast T1-weighted images for the evaluation of leptomeningeal diseases at 3T. AJNR Am J Neuroradiol.

[13] Mathews VP, Caldemeyer KS, Lowe MJ, Greenspan SL, Weber DM, Ulmer JL (1999). Brain: gadolinium-enhanced fast fluid-attenuated inversion-recovery MR imaging. Radiology.

[14] Lee EK, Lee EJ, Kim S, Lee YS (2016). Importance of contrast-enhanced fluid-attenuated inversion recovery magnetic resonance imaging in various intracranial pathologic conditions. Korean J Radiol.

